# Dr. John Lindesay Pearce

**Published:** 1934

**Authors:** 


### Obituary

### DR. JOHN LINDESAY PEARCE, M.C.,

### OF HAMBROOK, NEAR BRISTOL.

DR. JOHN LINDESAY PEARCE, who died suddenly on 10th April, 1934, at the early age of 52, was educated at Clifton College, of which he was a scholar, and subsequently at Edinburgh University, where he graduated M.B., Ch.B. in 1905. After holding resident appointments at Gloucester

Infirmary and at Dundee Royal Infirmary he practised at Finchingfield and at Great Bardfield in Essex until 1927, when he came to practise in Hambrook. During the Great War he served three and a half years with the R.A.M.C. in Gallipoli, Egypt and France : he was severely wounded in 1918 ; his military record was one of distinguished service, and he was awarded the Military Cross for devotion to duty. At Hambrook he was Vice-President of the local branch of the British Legion, and an enthusiastic supporter of the local cricket and football clubs. He took a keen interest in artistic matters, and was particularly well informed in questions relating to the Dutch School. He was a frequent attendant at medical meetings in Bristol. His sound professional knowledge and genial manner rendered him popular with all classes of society, and his loss has been deeply felt in the district. He leaves a widow and one daughter, to whom we extend our most sincere sympathy.

